# Experimental validation of state equations and dynamic route maps for phase change memristive devices

**DOI:** 10.1038/s41598-022-09948-6

**Published:** 2022-04-20

**Authors:** Francesco Marrone, Jacopo Secco, Benedikt Kersting, Manuel Le Gallo, Fernando Corinto, Abu Sebastian, Leon O. Chua

**Affiliations:** 1 Department of Electronics and Telecommunications, Politecnico di Torino, Torino, 10129 Italy; 2 IBM Research - Zurich, Rüschlikon, 8803 Switzerland; 3 Department of Electrical Engineering and Computer Sciences, University of California, Berkeley, 94720 CA USA

**Keywords:** Engineering, Nanoscience and technology, Physics

## Abstract

Phase Change Memory (PCM) is an emerging technology exploiting the rapid and reversible phase transition of certain chalcogenides to realize nanoscale memory elements. PCM devices are being explored as non-volatile storage-class memory and as computing elements for in-memory and neuromorphic computing. It is well-known that PCM exhibits several characteristics of a memristive device. In this work, based on the essential physical attributes of PCM devices, we exploit the concept of *Dynamic Route Map* (DRM) to capture the complex physics underlying these devices to describe them as memristive devices defined by a state—dependent Ohm’s law. The efficacy of the DRM has been proven by comparing numerical results with experimental data obtained on PCM devices.

## Introduction

PCM devices encode information on the phase configuration of a layer of material sandwiched between two metallic electrodes. This class of materials, typically compounds of Ge, Te and Sb exhibit a high electric conductivity in the crystalline phase and a much lower conductivity in the amorphous phase. In recent years, PCM devices have found application in stand-alone storage class memory^1^, embedded memory for edge computing^2^, reconfigurable electronics^3^ as well as neuromorphic^4,5^ and in-memory computing^6–8^.

**Figure 1:**
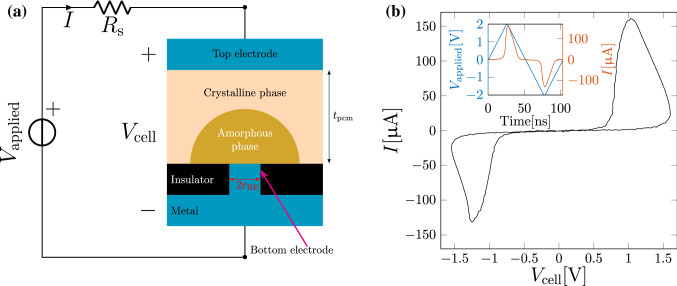
Mushroom-type PCM device and memristive characteristics. (**a**) Schematic representation of a mushroom-type PCM device operated with an integrated current compliance series resistor $R_\text {s}$. When the amorphous phase blocks the bottom electrode, the device is in a high-resistance state. The effective thickness of the amorphous region is denoted by $u_{\text {a}}$. $V_{\text {applied}}$ is the externally applied input voltage, *I* is the current flowing through the PCM device and $V_{\text {cell}}=V_\text {applied}-R_\text {s}I$ is the intrinsic voltage drop on the device. (**b**) The measured current-voltage relationship on the mushroom-type PCM device highlighting the pinched hysteresis loop characteristic of memristive devices.

A prototypical mushroom-type PCM^9^ device is schematically shown in Fig. 1a. A PCM device consists of a certain volume of phase change material sandwiched between the top and bottom metal electrodes. Applying current pulses to the device results in significant heating due to Joule heating. A RESET pulse refers to a current pulse which can melt a significant portion of the phase change material. When the pulse is stopped abruptly, the molten material quenches into the amorphous phase due to glass transition. In the resulting RESET state, the device will be in a high resistance state provided, the amorphous region blocks the bottom electrode. When a current pulse (typically referred to as the SET pulse) is applied to a PCM device in the RESET state, a part of the amorphous region crystallizes. Thus by modulating the size of the amorphous region by the application of suitable SET pulses, it is possible to achieve a continuum of resistance or conductance states. The resistance state achieved after the application of RESET or SET pulses can be deciphered by biasing the device with a small amplitude read voltage that does not disturb the phase-configuration.

PCM devices exhibit intriguing nonlinear dynamical behavior arising from a complex interaction among thermal, electrical and structural dynamics^10^. Accurate physical models exploiting integro-differential equations have been derived to capture the peculiar characteristic of the different operating conditions in PCM devices and then numerical analysis is essential to accurately describe the experimental observations^11^. However, it will be very interesting to describe the PCM device dynamics described as a memristive system as introduced in 1976 by S. Kang and L. O. Chua^12^. The pinched *current-voltage* loop characteristics shown by PCM devices under bipolar periodic input, as exemplified in Fig. 1b, clearly casts them in the class of memristive systems. In this work, our objective is to represent PCM devices via a state–dependent Ohm’s law, that is the Ohm’s law $v=R({\mathbf {x}},i)i$ linking voltage *v* across and current *i* through the two-terminal memristive device and a state equation $\dot{{\mathbf {x}}}=f({\mathbf {x}},i)$ governing the dynamics of the internal state variables, ${\mathbf {x}}$. The use of the memristive state–dependent Ohm’s law facilitates the visualization of the complex dynamics in PCM cells in terms of concepts such as Dynamic Route Maps (DRM) and the Power–Off–Plots (POP)^13^. When the state variable is scalar ($x\in {\mathbf {R}}$), the DRM represents the plot of *f*(*x*, *i*) in the plane $({\dot{x}},x)$; each DRM is parametrized by the input *i* and then a family of curves that span the whole plane $({\dot{x}},x)$. If $i=0$ then the DRM results into the POP and describe the long–term behavior of the memristive devices and their memory properties. Such a circuit-theoretic model is capable of accurately describing the electrical measurements and capturing the device physics in some key parameters. The use of DRMs, in addition, has been found to be a useful modeling method in recent years for similar devices. Ascoli *et al.* showed that such techniques can be exploited to investigate theoretically all the scenarios of the switching dynamics in memristive devices^14^. Subsequently, other works have shown how DRMs can be a powerful tool to model other devices such as ReRAMs, or of complex systems such as *Cellular Nonlinear Networks*^15,16^. In this work, we provide for the first time an experimental validation of these techniques for phase-change memory devices.

## Results

### Memristive state equations

**Figure 2:**
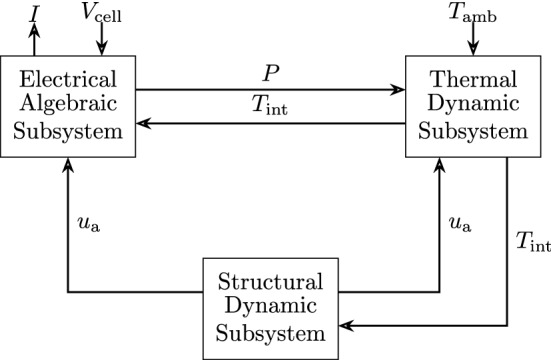
PCM dynamics. Block diagram illustration of the interplay between the electrical, thermal and structural dynamics in a PCM device. The system has two inputs $V_{\text {cell}}$ and $T_{\text {amb}}$ and one output *I*. $V_{\text {cell}}$ is the voltage drop on the PCM device. $T_{\text {amb}}$ denotes the ambient temperature at which the device is operated. *I* is the current flowing through the device. *P* is the electrical power dissipated by the device and is computed as the product between *I* and $V_{\text {cell}}$. $u_{\text {a}}$ is the effective thickness of the amorphous region and its evolution is determined by the crystallization dynamics. $T_{\text {int}}$ is the temperature at the amorphous-crystalline interface and its evolution is determined by the heat balance equation.

First, we will derive the memristive state equations based on the PCM device characteristics. A useful schematic illustration of the convoluted interconnections between the electrical, thermal and structural dynamics in PCM devices is reported in Fig. 2. The thickness of the amorphous region, $u_{\text {a}}$, is one of the key state variables. Another key variable is the temperature at the interface of the amorphous and crystalline regions denoted by $T_{\text {int}}$.

When an electrical pulse is applied to a PCM device, the voltage dropped across the device is denoted by $V_{\text {cell}}$. Depending on $V_{\text {cell}}$, the device could be in a low-field *OFF-state* or a high-field *ON-state*. In the *OFF-state*, the device conductance exhibits for increasing applied electric fields, linear, exponential and super-exponential dependence on $V_{\text {cell}}$^17^. In the *ON-state*, conduction through the amorphous phase is metal-like and the global flow of electrons in the PCM cell becomes dominated by the amorphous-crystalline Schottky barrier^18^. The rather complex transition between the OFF and ON states happens via the so called threshold switching event, the physical mechanism for which is being actively researched^11^. It is observed that threshold switching occurs once the current flowing though the device exceeds a fixed current, $I_\text {TH}$^11^ typically in the range of 10 $\mu$A.

A “write pulse” typically refers to an electrical pulse that results in a change in the phase-configuration. During the application of the write pulse, the device has to go to the *ON-state* since only in the *ON-state* is there sufficient power dissipation and structural dynamics to induce any meaningful change in $u_{\text {a}}$. If the write pulse amplitude is sufficiently large that the $T_{\text {int}}(u_{\text {a}})$ exceeds the melting temperature, $T_{\text {melt}}$, then $u_{\text {a}}$ increase in such a way that $T_{\text {int}}(u_{\text {a}}) = T_{\text {melt}}$. And if at this point, the pulse amplitude is abruptly brought to zero, then there is not enough time for crystallization and via the melt-quench process, a new amorphous region is created with the corresponding $u_{\text {a}}$. These type of pulses are referred to as RESET pulses and the amplitude of the write pulses are denoted by $I_\text {RESET}$.

In this work, we study write pulses that have sufficiently small amplitude (referred to as SET pules) such that $u_{\text {a}}$ reduces due to crystal growth. We first initialize the device with an appropriate RESET pulse to achieve an initial $u_{\text {a}}$. When such a write pulse is applied, the current flowing through the cell is denoted by *I* and the dissipated power is $P=V_{\text {cell}}I$. This results in an increase in $T_{\text {int}}$ which in turn will trigger a significant change in the rate of crystal growth. This alters the size of the amorphous region and in particular, $u_{\text {a}}$. In the *ON-state*, the resistance of the device is not indicative of $u_{\text {a}}$ as described earlier. Hence to decipher the phase-configurational information, the only way is to operate the device at the low-field *OFF-state*. This is typically referred to as the read operation. A more detailed analysis of the three key dynamical subsystems is presented next.

#### Thermal subsystem

**Figure 3:**
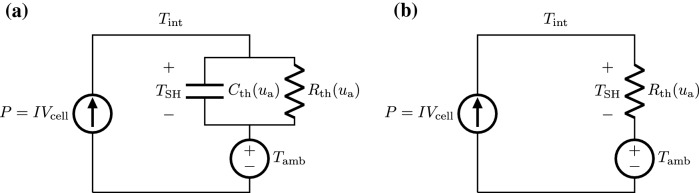
Thermal subsystem. (**a**) First order circuit model that takes into account the presence of $C_\text {th}(u_{\text {a}})$ (see Eq. (1)). (**b**) The simplified circuit model can be employed as described by Eq. (2) when the write pulse duration is substantially higher than $\tau _\text {th}$.

**Figure 4:**
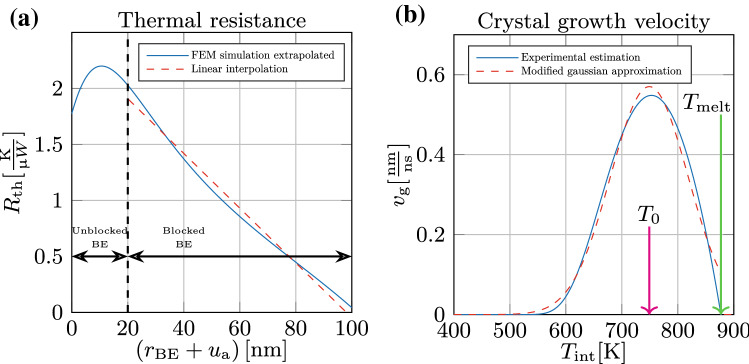
Effective thermal resistance and crystal growth velocity. (**a**) The effective thermal resistance of a mushroom-type PCM device as a function of the amorphous thickness $u_{\text {a}}$. This is obtained from Finite-element modeling that was also experimentally corroborated by Sebastian et al.^19^. Also shown is a linear approximation of the effective thermal resistance in the scenario where the bottom-electrode is fully blocked by the amorphous region (dashed-red line). (**b**) The experimentally measured crystal growth velocity $v_{\text {g}}(T_{\text {int}})$ as a function of the interface temperature $T_{\text {int}}$ based on Sebastian et al.^19^. Also shown is a Gaussian approximation (dashed-red line).

The thermal subsystem captures the evolution of the interface temperature, $T_{\text {int}}$ upon the dissipation of power, *P*, in the device upon the application of an electrical pulse (see Fig. 3). $T_{\text {int}}= T_{\text {amb}}+T_{\text {sh}}$ where $T_{\text {amb}}$ is the ambient temperature and $T_{\text {sh}}$ is the cell Joule self-heating temperature increment. $T_{\text {sh}}$ evolves according to,

1 $$\begin{aligned} \frac{d}{dt}T_\text {sh}(t)=\frac{1}{\tau _\text {th}}(R_\text {th}(u_\text {a}(t))P-T_\text {sh}(t)) \end{aligned}$$

where $\tau _\text {th}=R_\text {th}(u_\text {a}(t))C_\text {th}(u_\text {a}(t))$ is the thermal time constant and $R_\text {th}(u_{\text {a}}(t))$, shown in Fig. 4a, is the amorphous-thickness-dependent thermal resistance of the device which was estimated via finite element modelling (FEM)^19^. The thermal time constant was found to be on the order of a nanosecond^10^. Hence, for write pulses of duration larger than 10 ns, (1) can be reduced to an algebraic equation as

2 $$\begin{aligned} T_\text {sh}=R_\text {th}(u_\text {a})P \end{aligned}$$

and

3 $$\begin{aligned} T_\text {int}=T_{\text {amb}}+ R_\text {th}(u_\text {a})P \end{aligned}$$

Moreover, a good linear approximation of $R_\text {th}$ can be given, in the blocked bottom electrode condition, as

4 $$\begin{aligned} R_\text {th}(u_\text {a}(t))=-\kappa u_\text {a}(t)+R_{\text {th},0} \end{aligned}$$

where, $\kappa \approx -0.024\frac{K}{\mu W nm}$ and $R_{th,0}\approx 1.908\frac{K}{\mu W}$. Hence the interface temperature is assumed to instantly vary with the dissipated power as shown by the circuit representation in Fig. 3b.

#### Structural dynamic subsystem

The structural dynamic subsystem describes the evolution of the state variable, $u_\text {a}$. The evolution of $u_\text {a}$ is dictated by the temperature-driven crystallization dynamics given by,

5 $$\begin{aligned} \frac{d}{dt}u_\text {a}(t) = -v_\text {g}(T_\text {int}(t)) \end{aligned}$$

where $$u_{\text {a}}\in$$ [0,80]nm.

The crystal growth velocity, $v_{\text {g}}$, has a strong temperature dependence and was experimentally measured by Sebastian et al.^19^. A Gaussian approximation of $v_\text{g}(T_\text{int})$, shown in Fig. 4b, has also already been proposed by Secco et al.^20^, given by,

6 $$\begin{aligned} v_\text {g}(T_\text {int})=A_{v_\text {g}}exp\left[ -\left( \frac{T_\text {int}-T_0}{\sigma _T}\right) ^2\right] \end{aligned}$$

$A_{v_\text {g}} \approx$ 0.57 nm/ns, $T_0 \approx 749 \,{K}$ and $\sigma _\text {T} \approx 98$K. Note that, the changes of the material characteristics of the amorphous phase such as structural relaxation^21^ are not taken into account in this model. In the experimental data studied later, this effect was considered negligible due to the narrow time window over which the measurements are made.

#### Electrical subsystem

Next we consider the electrical subsystem which capture the electrical transport in a PCM device. The electrical transport is dominated by the more resistive amorphous region. As indicated earlier, there are two distinct regions of interest when it comes to the electrical subsystem. Here, we will delve a bit deeper into the *OFF-state* electrical transport that is relevant for the read operation. During read operation, there is minimal self heating and hence the temperature of the amorphous region can be assumed to be uniformly equal to $T_{\text {amb}}$.

More and more refined models have been developed over the years to explain how the two state variables $(u_\text {a},T_\text {amb})$ influence the electron conduction through the material in the *OFF-state*^22–25^. The 3D Poole-Frenkel^26–28^ emission of carriers from a two-center Coulomb potential was shown to best model in an unified manner the conduction in the amorphous phase-change material for a wide range of fields, *F*. According to this model, the density of free carriers under an applied field $F=V_{\text {cell}}/u_{\text {a}}$ at $T_{\text {amb}}$ is

$$\begin{aligned} n(F,T_{\text {amb}})=\frac{K}{2}\int _0^\pi exp( -\frac{E_\text {a}(T_{\text {amb}})-E_\text {PF}(F,\theta )}{k_\text {B} T_{\text {amb}}})\sin (\theta )d\theta \end{aligned}$$

where $E_\text {a}(T_{\text {amb}})$ is the interface temperature dependent activation energy

$$\begin{aligned} E_\text {a}(T_{\text {amb}})=E_\text {a,0}-\frac{aT_{\text {amb}}^2}{b+T_{\text {amb}}} \end{aligned}$$

and $E_\text {PF}(F,\theta )$ is the the energy barrier lowering between two adjacent potential wells due to the Poole-Frenkel effect and is computed as

$$\begin{aligned} E_\text {PF}(F,\theta )=-\max _\text {r}\Phi (r,\theta ,F) \end{aligned}$$

where $\Phi (r,\theta ,F)=-qFr\cos (\theta )-\frac{\beta ^2}{4q}(\frac{1}{r}+\frac{1}{s-r})+\frac{\beta ^2}{qs}$ is the electrical potential profile, *s* is the distance between two defect centers and $\beta =\frac{q^2}{\sqrt{q\pi \epsilon _r\epsilon _0}}$.

Conductivity of the amorphous phase per unit of area can then be computed as

$$\begin{aligned} \sigma (F,T_{\text {amb}})=q\mu (F)n(F,T_{\text {amb}}) \end{aligned}$$

where $\mu (F)$ is the field dependent carrier mobility given by $\mu (F)=\frac{\mu _0}{\sqrt{1+(\mu _0F/v_\text {sat})^2}}$. The current density $j(F,T_{\text {amb}})$ can be computed as $j(F,T_{\text {amb}})=\sigma (F,T_{\text {amb}})F$. and the current flowing through the cell can be found by simply multiplying the current density $j(F,T_{\text {amb}})$ times the effective bottom electrode contact area *A* which is calculated through the effective bottom electrode radius and not the physical radius of the heater, $I(F,T_{\text {amb}})=Aj(F,T_{\text {amb}})$. The model parameters are reported in Table 1.

**Table 1:** Parameters associated with the electrical subsystem.

Parameter	Value	Unit
$K\mu _\text {0}$	$10^{22}$	m$^{-1}$V$^{-1}$s$^{-1}$
$E_\text {a,0}$	225	meV
*a*	600	$\upmu$eVK$^{-1}$
*b*	800	K
*s*	2.4	nm
$v_\text {sat}$/$\upmu _\text {0}$	50	V$\upmu$m$^{-1}$
$\epsilon _\text {r}$	10	

### State dependent Ohm’s law

**Figure 5:**
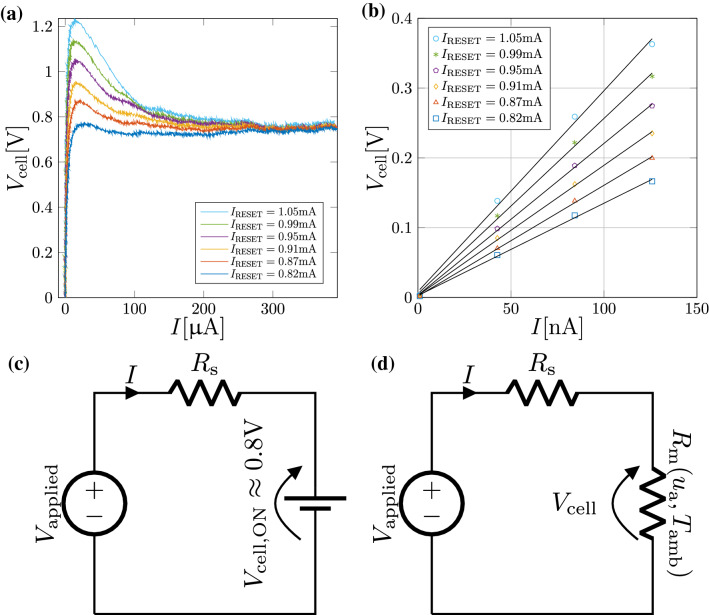
Electrical subsystem. (**a**) The measured $I-V_\text {cell}$ characteristics of the device when write pulses of different reset currents $I_\text {RESET}$ are applied. It can be seen that beyond $I=$ 100 $\mu$A, $V_{\text {cell}}$ plateaus to approx. 0.8 V. (**b**) The low-field $I-V_{\text {cell}}$ characteristics are measured after the application of each write pulse. An almost linear behavior is observed. Moreover, the resistance progressively increases with increasing $I_\text {RESET}$. (**c**) During the application of the write pulse, the PCM device can be modeled as an ideal voltage generator $V_\text {cell,ON}$. (**d**) During read when probing the phase-configuration of the device, the PCM device can be modeled as a state-dependent linear resistor.

To summarize, during the application of write pulses, when the device is biased at high field, it goes into a very low resistance state independent of the state variable $u_{\text {a}}$. As shown in Fig. 5a, the voltage drop $V_{\text {cell}}$ is confined to a narrow voltage band around $V_\text {cell,ON}\approx$ 0.8 V. This enables the simplification of the complex conduction mechanism to a simple nonlinear current-controlled resistor whose behavior for high enough currents can be well approximated by an ideal voltage generator of value $V_\text {cell,ON}$.

However, during read operation, we retrieve the phase-configurational information by application of a low amplitude current pulse. Under the hypothesis of fully blocked bottom electrode ($u_\text {a}>0$) and very *low-field* ($F=\frac{V_\text {cell}}{u_\text {a}}\rightarrow 0$), the voltage can be well approximated by a linear relation $V_\text {cell}=R_\text {m}(u_\text {a},T_{amb})I$, as shown in Fig. 5b, where the memristance $R_\text {m}(u_\text {a},T_\text {amb})$ is given by

7 $$\begin{aligned} R_\text {m}(u_\text {a},T_\text {amb})=K^{'}u_\text {a} \exp \left( \frac{E_\text {a}(T_\text {amb})}{ k_\text {B} T_\text {amb}}\right) \end{aligned}$$

being $k_\text {B}$ the Boltzmann constant, $E_\text {a}(T_\text {amb})$, the temperature-dependent activation energy and $K^{'}=\frac{1}{\pi r^2_\text {BE}q_\text {e}K\mu _0}$. This expression for the memristance $R_\text {m}(u_\text {a},T_\text {amb})$ can readily be derived from the Taylor series expansion of the Poole conduction model.

These aspects are summarized in the following state-dependent Ohm’s Law,

8 $$\begin{aligned} V_\text {cell}\approx {\left\{ \begin{array}{ll} R_\text {m}(u_{\text {a}},T_{\text {amb}})I & I \ll I_\text {TH}\\ 0.8V & I \gg I_\text {TH} \end{array}\right. } \end{aligned}$$

where the well-above-threshold-switching model ($I\gg I_\text {TH}$), depicted with circuital symbolism in Fig. 5c, is intended to capture the cell power dissipation during the write phase. The interface temperature, $T_{\text {int}}$, is in turn a function of the dissipated power and the instantaneous $u_{\text {a}}$ (see Equation (3)). The $T_{\text {int}}$ in turn determines the crystal growth velocity which drives the structural dynamics and the evolution of $u_{\text {a}}$ (see Equation (5)). Armed with these equations, we can explore the dynamic route maps of PCM devices. Finally, the well-below-threshold-switching model ($I\ll I_\text {TH}$), portrayed with circuital symbolism in Fig. 5d gives a good approximation for the read phase where it is possible to observe the $u_{\text {a}}$ state variable.

### Dynamic route maps

**Figure 6:**
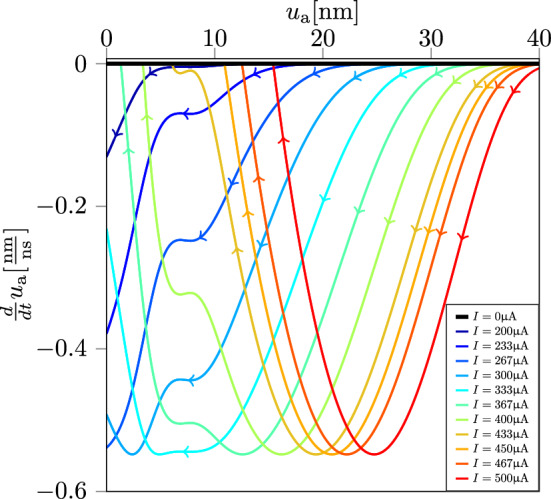
Dynamic route map for PCM. The computed dynamic route map for the PCM device corresponding to write pulses of varying amplitudes.

Based on the state equations described in earlier sections, we performed a set of simulations to obtain the DRMs for the PCM device (see Fig. 6). The SET write pulse amplitude was varied from $I=$ 200 $\mu$A to 500 $\mu$ A. It can be seen that there is a significant dependence on the current amplitude. For example, for SET current amplitudes equal to or larger than 333 $\mu$A, each route, when traversed, leads to a distinct nonzero equilibrium point. Whereas, for currents lower than 333 $\mu$A, $u_{\text {a}}$ becomes zero. The reason is that, for the larger RESET currents, as $u_{\text {a}}$ reduces, $T_{\text {int}}$ increases and eventually, it becomes equal to $T_{\text {melt}}$ at which point crystal growth rate becomes zero. However, for lower RESET currents, $T_{\text {int}}$ remains lower than $T_{\text {melt}}$ resulting in a complete erasure of the amorphous region.

**Figure 7:**
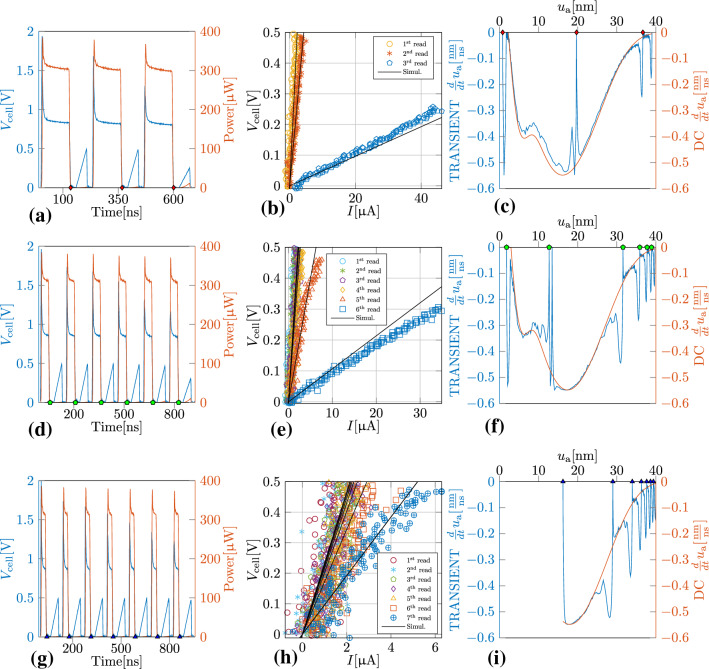
Experimental validation of DRM. Panels (**a**–**c**), (**d**–**f**) and (**g**–**i**) correspond to three separate experiments to measure the DRM. (Left column) Experimentally measured $V_{\text {cell}}$ and the corresponding dissipated electrical power, *P*. Each square-shaped high–current write pulse is interleaved with a below threshold triangular read pulse (see Methods for more details). Between experiments, the key difference is the number and duration of the write pulses. (Center column) The $I-V_{\text {cell}}$ characteristics corresponding to the read pulses are shown. The effective amorphous thickness, $u_{\text {a}}$, is assumed to be directly proportional to the slope of these curves as per Eq. (7). (Right column) Based on the power signals shown on the left column, the $u_{\text {a}}$ estimates based on the $I-V_{\text {cell}}$ characteristics shown in the center column and the memristive state equations, experimental DRMs are obtained (blue traces). Also shown are the DRMs based on the steady-state values of the power signals (red traces).

A key goal of this work is to experimentally measure the DRMs. To that end, we performed a set of experiments that involve applying SET pulses to the PCM device with a certain programming current but with varying pulse duration (see Fig. 7). In the first experiment, we applied 3 pulses each of duration 121 ns(see Fig. 7a). After each pulse, we apply a triangular pulse with low amplitude to probe the low-field resistance and subsequently, to estimate $u_{\text {a}}$. We repeated this experiment two more times with more number of pulses with smaller duration. The smaller duration pulses facilitate more frequent probing of $u_{\text {a}}$ during its evolution. Based on the experimental voltage and current traces, the dissipated power was calculated which in turn was used to calculate $\frac{du_{\text {a}}}{dt}$. In this manner, we could experimentally measure the DRM for the PCM device. As expected, all three experiments yielded comparable DRMs since the pulse amplitude was kept constant.

The DRMs can be used as a tool to tune the SET programming current to achieve a certain desired SET state. This is clear when looking at Fig. 6, starting from an high resistance *RESET* state associated with $u_a(t_0)$ $\approx$ 50 nm, the designer can choose to reach three different low resistance states by tuning the SET programming current. This in turn can be exploited to design programmable circuits such as programmable amplifiers and tunable filters. In the latter case, the knowledge about how the equilibria of $u_\text {a}$ depend on the programming set current $I_\text {SET}$ allows to precisely tune the position of zeros and poles in a completely analog fashion.

**Figure 8:**
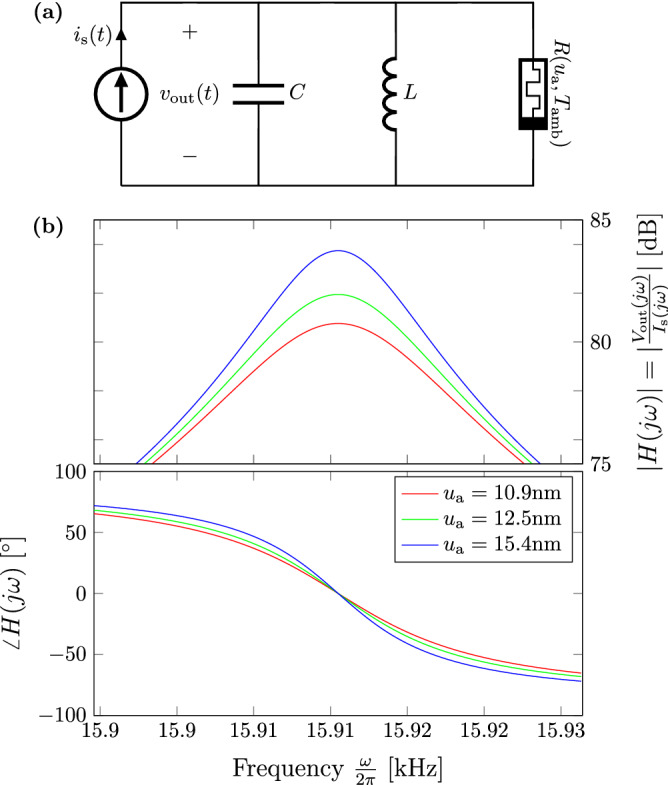
(**a**) Application in re-configurable electronics. Passive tunable RLC passband filter exploiting a PCM device in its read region via $R_{\text {m}}(u_{\text {a}},T_{\text {int}})$. (**b**) Bode plots of the transfer function $H(j\omega )$ parametrized on the final resistance values reached by following the DRMs in Fig. 6.

Let us consider a simple example where a PCM device is embedded into a passive (resistor-inductor-capacitor) RLC passband filter as reported in Fig. 8. Let us assume the input to be a small signal current $i_\text {s}(t)$ (e.g. current coming from a sensor) and to be measuring the voltage drop $v_\text {out}(t)$ on the parallel RLC. For fixed values of the inductance *L* and capacitance *C*, the quality factor $Q = R_{\text {m}}(u_{\text {a}},T_{\text {int}})\sqrt{\frac{C}{L}}$ can be tuned to a desired value following the corresponding DRM in Fig. 6. Considering the Laplace-transformed port variables $I_{\text {s}}(j\omega ) = {\mathcal {L}}(i_{\text {s}}(t))$ and $V_{\text {out}}(j\omega ) = {\mathcal {L}}(v_{\text {out}}(t))$, Bode plots of the transfer function $H(j\omega ) = \frac{V_{\text {out}}(j\omega )}{I_{\text {s}}(j\omega )}$ are reported in Fig. 8**b** parameterized on the final values reached by following the DRMs in Fig. 6. With *C* and *L* being fixed in the example to 1 $\mu$F and 0.1 mH respectively, it is evident how the quality factor, *Q*, can be modulated by first resetting the PCM cell and then setting it back with a constant SET current I. Similar applications can also be found in areas such as neuromorphic computing where the goal would be to tune synaptic weights in a neural network^29^.

## Conclusion

Phase-change memory is a highly promising post-CMOS technology that is finding applications as non-volatile memory, compute elements for in-memory and neuromorphic computing and also as components of reconfigurable electronic circuits. In this paper, we have developed a memristive state-dependent Ohm’s law to describe the dynamics of PCM devices when SET write pulses are applied. This was based on the observation that the PCM dynamics is an intricate interconnection of electrical, thermal and structural dynamics. We developed a simulation model based on published literature as well as newly obtained experimental measurements on a mushroom-type PCM device. We also computed the dynamic route maps for the device upon the application of SET write pulses after initializing the devices to a certain RESET state. Finally, we experimentally measured the DRM with carefully executed experiments involving the successive application of short write pulses interspersed with read pulses. Given, the ultra-small timescales associated with the crystallization kinetics, this is a remarkable experimental feat and has helped establish an accurate memristive model to describe PCM dynamics. One of the crucial information drawn from the DRMs for PCM is the directed design of SET pulses to drive the thickness of the amorphous region and the associated low-field resistance. By exploiting the knowledge gained on the temporal evolution of the amorphous thickness, suitable SET pulses can be chosen to modulate dissipation in analog electronic circuits. As a case study we presented a PCM–based analog filter. This work is a significant step towards the inclusion of memristor–based PCM model in automatic design tools for programmable analog circuits and also for tunable synaptic elements in neuromorphic circuitry, overcoming the limitations posed by the complex dynamics of these elements.

## Methods

### Phase-change memory devices

The mushroom-type PCM devices used in the experiments were fabricated in the 90 nm CMOS technology node with the bottom electrode created via sublithographic key-hole process. The phase change material is doped Ge$_2$Sb$_2$Te$_5$. The dopant is primarily intended to enhance the endurance of the PCM cell. The bottom electrode (BE) has a radius of $\approx$ 20 nm and length of $\approx$ 65 nm. The phase change material is $\approx$ 100 nm thick and extends to the top electrode (TE), the radius of which is $\approx$ 100 nm. In the same chip with the PCM devices, there is an integrated series resistor per device denoted by $R_{\text {s}}$ which has a value of approx. 5.7 k$\Omega$.

### Experimental setup

For measurements that involve the application of fast pulses, an arbitrary waveform generator (AWG) was employed to apply the desired input stimulus and a Digital Storage Oscilloscope (DSO) to measure the current *I* and the applied voltage $V_{\text {applied}}$. The DSO acquired all the signals at a sampling frequency $f_{\text {s}}=$ 2.5 GHz. An Agilent 81150A Pulse Function Arbitrary Generator was used and a Tektronix TDS3054B oscilloscope for AC voltage and current measurements. For slower steady-state $I-V$ measurements, a Keithley 2400 SMU was used. $V_{\text {applied}}$ is directly measured by the DSO from the AWG. The resulting current that flows through the device is measured using a simple current to voltage conversion circuitry. Given the length of the wires ($\approx$ 60 cm) and the involved time scales, the phase shift of current with respect to the $V_{\text {applied}}$ signal has to be taken into account. The delay was estimated to be $\approx$ 6.4 ns.

### Experimental measurement of DRM

Before performing each of the three measurements, the cell was reset with a 950 $\mu$A amplitude square pulse of 1 $\mu$s duration and sharp leading and trailing edges of 7.5 ns each. The reset was intended to create an amorphous dome of effective thickness, $u_{\text {a}}\approx$ 40 nm. This initial $u_{\text {a}}$ was estimated by fitting the $I-V_{\text {cell}}$ characteristics to a transport model proposed by Ielmini and Zhang^30^. A train of interleaved write and read pulses were applied 20 $\mu$s after applying the reset pulse. Each write pulse with 7.5 ns leading and trailing edges had an amplitude of $\approx$ 2.93 V and a variable duration ranging between 25 ns and 121 ns). Each write pulse was followed, after 25 ns, by a read ramp voltage pulse of 0.5 V peak value and 50 ns duration. The reading voltage ramps had a 7.5 ns trailing edge and were followed by a new writing pulse after 25 ns.
